# Children and unintentional firearm death

**DOI:** 10.1186/s40621-015-0057-0

**Published:** 2015-10-12

**Authors:** David Hemenway, Sara J. Solnick

**Affiliations:** 1 Harvard School of Public Health, 677 Huntington Avenue, Boston, MA 02115 USA; 2 Department of Economics, University of Vermont, Burlington, VT USA

**Keywords:** Firearms, Children, Unintentional gun deaths, Gun accidents

## Abstract

**Background:**

Children in the United States are at far greater risk of unintentional gun death than children in other developed countries. The relative figures may even be worse since the estimates for US child unintentional gun deaths are derived from the Vital Statistics which have been shown to be underestimates. No study has used a national data system to investigate the circumstances of fatal child gun accidents.

**Methods:**

We use data from the National Violent Death Reporting System for 16 states from 2005 to 2012. We examine the cases of unintentional gun death involving children in five age groups, 0–1, 2–4, 5–10, 11–12, and 13–14, where the child was either the victim or shooter.

**Results:**

We estimate that there were 110 unintentional firearm deaths to children 0–14 annually in the U.S. during this 8 year time period, 80 % higher than reported by the Vital Statistics. The victims were predominantly male (81 %). Approximately two thirds of the shootings were other-inflicted, and in 97 % of those cases the shooter was a male. The typical shooter in other-inflicted shootings is a brother or friend. Indeed, children aged 11–14 are often shot in the home of friends. The large majority of children are shot by other children or by themselves. It is rare for a child accidentally to be shot by or accidentally to shoot an adult who is not a family member.

**Conclusions:**

Our study highlights the fact that unintentional firearm death to children is a problem of children shooting children and thus the importance of keeping guns away from children, their siblings, and their friends.

## Background

Children ages 0–14 in the United States have far higher rates of unintentional firearm death than children in other developed countries—on the order of 10 times higher (Richardson and Hemenway 2011). This ratio is most likely an underestimate since the US data it relies upon come from death certificates. It has been shown that many unintended shootings of children in the U.S. (e.g., two children were playing with a gun they thought was unloaded) are classified in the Vital Statistics as homicides (Schaechter et al. 2003; Barber and Hemenway 2011; Luo and McIntire 2013).

While Vital Statistics data are accurate for examining total firearm deaths, there is serious misclassification between types (e.g., homicides and accidents). A study examining the accuracy of data systems for unintentional firearm fatalities found the Vital Statistics system missed 38 % of the true cases (mostly gun accidents to children that were classified as homicides) and 42 % of the cases reported were false positives (mostly gun deaths to adults that were of undetermined intent). By contrast, the accidental gun deaths reported in the National Violent Death Reporting System (NVDRS) had a Positive Predictive Value of 99 % (Barber and Hemenway 2011).

This article uses NVDRS data to examine unintentional firearm deaths to children. NVDRS has another great advantage over Vitals data in that it provides more complete information, including a narrative of the event.

The literature on unintentional firearm deaths of children is very small. Older studies tended to be hospital-based case studies (Heins et al. 1974; Ordog et al. 1988) or used data from a single county or state (Wintemute et al. 1987; Grossman et al. 1999; Cherry et al. 2001). A recent analysis by an advocacy group gathered information from news reports for the entire nation for one year (Moms Demand Action 2014). As far as we can tell, only one other study on unintentional firearm fatalities used NVDRS data (Hemenway et al. 2010) and that study did not focus on children. No one has analyzed the NVDRS data focusing on child unintentional firearm fatalities.

Except for anecdotes and case studies, there do not seem be any studies of children as shooters in unintentional firearm deaths—except in the description of their role as accidental shooters in the deaths of other children. This study is the first to use NVDRS to analyze the incidence and characteristics of children as shooters in all unintentional gun deaths.

This study has four aims: (1) to estimate the annual number of unintentional firearm deaths to children aged 0–14 in the United States; (2) to describe the circumstances of such events; (3) to describe the shooters in such events; and (4) to provide some information about child shooters in all unintentional firearm deaths, including when children shoot adolescents and adults.

## Methods

We use data on child violent death from states reporting to the National Violent Death Reporting System (NVDRS) for all years 2005–2012. Sixteen of the reporting states met that criteria (Alaska, Colorado, Georgia, Kentucky, Maryland, Massachusetts, New Jersey, New Mexico, North Carolina, Oklahoma, Oregon, Rhode Island, South Carolina, Utah, Virginia, and Wisconsin). Ohio only began providing data in 2010 and was excluded.

The NVDRS is a state-based surveillance system that links data from the death certificate, law enforcement records, and coroner/medical examiner records on deaths due to suicide, homicide, legal intervention, unintentional firearm injury, and firearm fatalities of unknown intent (Paulozzi et al. 2004, Hemenway et al. 2010, Barber et al. 2013). The records are incident-based and include information on the persons (victims and perpetrators), weapons, and circumstances involved. In addition to the coded data, the abstractor writes incident narratives summarizing the findings from the coroner/medical examiner records and the law enforcement records. Data are collected and linked at the state level, stripped of personal identifiers, and forwarded to the Centers for Disease Control and Prevention (CDC). Data for this study come for the NVDRS Restricted Access Dataset (RAD). This study was reviewed by the Harvard Human Subjects Institutional Review Board and deemed exempt since it uses only publicly available data. 

The authors of this paper reviewed every violent death report that was classified as an unintentional firearm death involving a child aged 0–14 as either victim or shooter. We also read every suicide (*n* = 574), homicide (*n* = 2143) and killing with undetermined intent (*n* = 268) of children aged 0–14. We also read every homicide classified as having a child perpetrator (*n* = 146). The narratives were read by both authors, and in the very few cases of disagreement, we met and reached agreement.

We only re-categorized cases where the evidence was clear that the shooting was unintentional (e.g., the narrative stated the shooter was “playing with the gun” or “thought the gun was unloaded”) and there was no evidence of a precipitating argument or anger between the shooter and victim.

For information on whether the shooting took place at the home of a friend, and for whether it involved hunting, we relied on the case narratives. In the hunting category, we included deaths occurring not only during the hunt itself, but in the preparation for the hunt and restoring the firearms following the hunt.

A priori, we established age groups as follows: ages 0–1 (infants); 2–4 (pre-school); 5–10 (K-5); 11–12 (pre-teen) and 13–14 (pre-adolescent).

## Results

NVDRS reported 191 cases of unintentional firearm fatalities of children aged 0–14. Eight (8) of these cases were sudden infant deaths not involving a firearm, reducing the count to 183. We determined that none of the cases classified as suicides were clearly unintentional; 40 of the cases classified as a homicide shooting were actually unintentional; and 6 of the undetermined shootings were clearly unintentional. We thus estimate that there were 229 unintentional firearm fatalities of children (aged 0–14) in the 16 states for the eight year period (Table 1). That is 1.8 deaths per million life years.

**Table 1:** Victims (0–14) of unintentional firearm fatalities, 2005–2012, 16 states

Age	# of cases	Years of exposure (000)	Deaths/ million years	# Male	# Female	% Male	# Self-inflicted	Other-inflicted	Unk	% Other (w/o unk)
0–1	7	17,110	0.4	2	5	29 %	1	6	0	86 %
2–4	42	25,757	1.6	37	5	88 %	27	11	4	29 %
5–10	69	51,407	1.3	52	17	75 %	18	46	5	72 %
11–12	51	17,464	2.9	40	11	78 %	13	35	3	73 %
13–14	60	17,623	3.4	54	6	90 %	12	43	3	78 %
0–14	229	129,361	1.8	185	44	81 %	71	141	17	66 %

The child population of these 16 states is 26 % of the total US child population, and Vital Statistics data for the years 2005–2012 show that the number of total firearm deaths for the children in these 16 states represents 26 % of the total firearm deaths for the US child population. We thus estimate that the total number of unintentional firearm deaths to children in the US during this eight year period was 229/.26 = 881, or 110 per year.

For the 16 states in this study, the rate of unintentional firearm death was highest among the 11–14 year olds. Over four fifths (81 %) of the victims were male; for the 13–14 year olds, 90 % were male (Table 1).

Nineteen per cent (19 %) of the deaths occurred at the house of a friend; only one child under age 11 was killed at a friend’s house (Table 2). Eleven percent (11 %) of the deaths involved hunting (including pre-and post-hunt injuries) (Table 2).

**Table 2:** Victims (0–14)

Age	# of cases	# hunting	% hunting	# at Friends house	% at Friends house
0–1	7	1	14 %	0	0 %
2–4	42	0	0 %	0	0 %
5–10	69	10	14 %	1	1 %
11–12	51	8	16 %	16	31 %
13–14	60	7	12 %	26	43 %
0–14	229	26	11 %	43	19 %

Of the 229 cases, in 17 it was not reported whether or not the victim shot himself or was shot by someone else. Excluding these unknowns, two thirds (66 % or 141/212) of the shootings were other-inflicted; only in the 2–4 year old age group were most shooting deaths self-inflicted (Table 1).

In 18 of the other-inflicted shootings, the relationship of the shooter to the victim was not reported. Excluding these unknowns, the shooters in other-inflicted deaths were nearly always either family members (61 % or 75/123) or friends (30 %; 37/123) (Table 3). In other words, family or friends were the shooters in over 90 % of all the other-inflicted shootings.

**Table 3:** Characteristics of other-inflicted shooters of children aged 0–14

Victim age	# of cases	Male	Female	% male	Brother	Father	Other family	% Family	Friend	Other
0–1	6	5	0	100 %	1	3	1	100 %	0	0
2–4	11	8	0	100 %	5	1	3	90 %	0	1
5–10	46	34	1	97 %	16	3	12	79 %	5	3
11–12	35	29	1	97 %	11	3	7	66 %	8	3
13–14	43	36	1	97 %	5	0	4	24 %	24	4
0–14	141	112	3	97 %	38	10	27	61 %	37	11

Excluding unknowns, the percentage of other-inflicted shooters who were a family member fell with the age of the victim (from 100 % of the 0–1 year old victims to 24 % of the 13–14 year old victims) (Table 3) while the percentage of other-inflicted shooters who were a friend rose (from 0 to 65 %). Among family, brothers were commonly the shooters accounting for over half of the family-related deaths and 31 % of all unintentional other-inflicted deaths. Eight percent (8 %) of all other-inflicted shooters were the father. Ninety-seven percent (97 %) of the other-inflicted shooters were male.

The age of 20 % (*n* = 28) of the other-inflicted shooters was not reported. Excluding these unknowns, about two thirds (65 %) of the other-inflicted shooters were children aged 0–14 (Table 4). Counting children who shot themselves, 78 % (144/184) of the shooters were aged 0–14. Among the shooters older than age 14, half were older teenagers (aged 15–19), usually aged 15–16 (friends of the predominantly 13–14 age victims), and half were adults, usually immediate family members. Of the 40 older (age 15+) perpetrators, over 57 % were family members (Table 4).

**Table 4:** Other-inflicted shooters of children aged 0–14

Age of Shooter	# of cases
0–1	0
2–4	3
5–10	25
11–12	22
13–14	23
15–19	20
20+	20
Unknown	28
Total	141

Of the 40 perpetrators over age 14, there were 23 family members, 7 friends, 3 acquaintances, 1 police, 2 known other, 1 stranger, 3 unknown

Children not only shoot other children, but they also sometimes shoot older teenagers and adults. Reading all the cases classified as homicides when children shot older teens and adults, we found only 2 instances where the case should have been considered an accident. Including those meant there were 21 cases where a child accidently shot and killed someone over age 14 (Table 5). Twelve (57 %) of these older victims of child shooters were older teenagers, and 9 (43 %) were adults. Among the 21 older victims of child shooters, 6 were brothers, 4 parents, 2 other family, 6 friends, and 3 others.

**Table 5:** Characteristics of Children (0–14) as Shooters in all unintentional firearm fatalities

Age of shooter	# of cases	Male	% male	Shoot self	Victim other children	Victim 15–19 year olds	Victim 20+
0–1	1	1	100 %	1	0	0	0
2–4	30	27	90 %	27	3	0	0
5–10	45	34	76 %	18	25	0	2
11–12	43	37	86 %	13	22	3	5
13–14	46	45	98 %	12	23	9	2
0–14	165	144	87 %	71	73	12	9

Of the 21 victims over age 14, 6 were brothers, 4 parents, 2 other family, 6 friends, 1 stranger, 1 acquaintance, 1 known other

To give a flavor for the circumstances in which children are killed unintentionally, we provide an example of a child victim of each age:

A 0 year old (9 months) female was shot by her father. While he was picking up his daughter, his gun fell out of his holster and when he grabbed for it, it went off.

A 1 year old (20 months) female was shot by her 9 year old brother who was playing in his room with a 410 gauge shotgun. He slipped on a toy and pulled the trigger by accident.

A 2 year old male was at a relative’s home and picked up a gun that was left on top of a microwave oven.

A 3 year old male was accidently shot by his father who was cleaning his .50 caliber handgun, which he thought was unloaded.

A 4 year old female was shot by her 11 year old brother who found the gun in a relative’s bedroom.

A 5 year old female was in her home with another child when they found a rifle in the closet. They began playing with the weapon and it went off.

A 6 year old male was unintentionally shot in the head after his twin brother decided to take a rifle off a gun rack in their bedroom.

A 7 year old was shot unintentionally in the back by his 10 year old brother. They had been with their father when the victim ran in front of his brother while the latter was shooting at a deer.

An 8 year old male was shot by his 12 year old brother. They had been playing with a handgun when the trigger unintentionally engaged.

A 9 year old male was accidentally shot by his 13 year old cousin in their grandparent’s home. The victim found a gun in a drawer in his grandfather’s bedroom. The gun reportedly was not working. Victim and suspect were playing with the gun when it went off.

A 10 year old male was accidently shot by his father in the woods when they were hunting. The father slipped while carrying a rifle as they descended a hill.

An 11 year old male was shot in the chest by his brother. They were at a relative’s house and found the gun in the entryway.

A 12 year old male was at his 12 year old friend’s house when the latter produced his father’s semi-automatic pistol and began showing it to the victim.

A 13 year old died of an accidental shotgun wound to the face at his friend’s residence. Suspect had retrieved the step father’s shotgun and was showing it to the victim.

A 14 year old female was shot at home by her 10 year old brother who was playing with the firearm.

## Discussion

We estimate that there are 110 children (age 0–14) killed unintentionally with guns each year in the United States (1.8 deaths per million life years). This number is 80 % higher than the figure derived from Vital Statistics (WISQARS) (1.0 deaths per million life years)—a figure which has been shown to be a substantial underestimate (Schaechter et al. 2003; Barber and Hemenway 2011; Luo and McIntire 2013; Moms Demand Action 2014) in large part due to its mis-classification of gun accidents as homicides. As expected, the Vital Statistics data are particularly lower for the age group 5–14, where more shootings are other-inflicted. Many cases are mis-classified as homicides in the Vital Statistics simply because the shooter pulled the trigger intentionally, even though he believed the gun was unloaded. While our estimates of deaths per million years are equal to the Vital Statistics for victims aged 4 years and under, our estimates are approximately double those of the Vital Statistics for victims aged 5–10, 11–12 and 13–14.

Even within the NVDRS, we concluded that some cases needed to be reclassified. Our reclassification of cases, particularly from homicide to unintentional increased our estimate from 88 unintentional child firearm fatalities per year to 110. Except for the 8 NVDRS cases that did not involve a firearm and were sudden deaths to infants, we did not find any case classified as an unintentional shooting that we believed should have been re-classified as another type of death.

To extrapolate our results to obtain a national estimate, we used the fact that our sample states contained both 26 % of the child population of the US and accounted for 26 % of the children killed by firearms in any manner (i.e., homicides, suicides, accidents and undetermined). As a sensitivity analysis, we also examined our 16 states in terms of household gun ownership. Our sample contained relatively populous states with high levels of household gun ownership (e.g. Georgia and North Carolina) and low levels (e.g., New Jersey, Massachusetts). Using household gun ownership data from a recent study (Kalesan et al. 2015) we found that households in our 16 sample states had the same level of gun ownership as households in the other 34 states; using data from the Behavioral Risk Factor Surveillance System for 2001, 2002 and 2004, we estimate that households in our 16 states had slightly higher levels of gun ownership. Using these latter estimates to extrapolate, our estimate would fall to somewhat over 100 unintentional gun deaths per year to children.

Whatever the precise number of unintentional firearm deaths, these are only the tip of the iceberg in terms of all accidental firearm injuries. It is estimated that for every death, there are 10–20 non-fatal unintentional woundings (Vyrostek et al. 2004; Barber et al. 1998).

We find that vast majority of children who die from unintentional firearm deaths either have shot themselves or have been shot by other children or by their own parents. A recent claim that “about two thirds of accidental gun deaths (to children younger than 10) are not shots fired by other little kids but rather by adult males with criminal backgrounds” (Lott 2013) is assuredly wrong. We find that for children aged 10 and under, of the 118 cases (including 9 where no information was provided on the shooter), in 39 % the child shot him/herself, and in the 63 cases which were definitely other-inflicted, the shooter was a brother in 35 % of the cases, another family member in 37 % of the cases, and a friend in 8 % of the cases. In only 4 cases was the shooter known to be neither a family member nor friend (one was a police officer).

We find that 11 % of all child deaths are hunting-related. Of these, 40 % were self-inflicted, 40 % of the victims were shot by family members, and 20 % by friends. Our hunting estimate may be higher than most because we include deaths occurring not only during the hunt itself, but in the preparation for the hunt and restoring the firearms following the hunt.

Our results indicate that there is danger in children visiting another home which contains a firearm. However, the danger does not seem to be a substantial one until the child turns 11. In our data set, only one child aged 10 or under was killed accidently at a friends’ house; by contrast, for the 11–14 year old age group, 39 % of accidental gun deaths occurred at the home of a friend. For this older age group a parent may want to ask the parents whose home their child is visiting about the accessibility of firearms (Johnson et al. 2012); asking seems less important for younger children. Perhaps the disparity is due to younger children being better supervised by adults in the households they visit, or younger children being less tempted to play with guns.

Our findings are consistent with other studies of unintentional gun deaths to children. For example, we find that males are the likely victims (81 %), and males are even more likely to be the shooter in other-inflicted shootings of children (97 %) (Wintemute et al. 1987: Moms Demand Action 2014). We also find that in the 2–4 year age group, the large majority of gun deaths are self-inflicted, while for other ages, 0–1, 5–14, deaths are far more likely to be other inflicted (Wintemute et al. 1987; Moms Demand Action 2014). Consistent with these other studies, we find that the 2–4 year olds have a higher risk of unintentional gun death than 5–10 year olds.

Our study appears to be the first to examine the situations in which children unintentionally shoot and kill older adolescents and adults as well as other children. While the occasional toddler who shoots and kills her parent makes news, this scenario is rare. We found only 1 case of a child 10 years old or younger unintentionally shooting and killing an adult.

Only a small percentage of child victims are shot by adults, and an even smaller percentage of child shooters kill adults. There are thus somewhat more child victims than child shooters. It is important to note that shooting someone, even unintentionally, may be a substantial risk factor for subsequent mental health problems, including PTSD and alcohol abuse (Connorton et al. 2011).

For most of the cases, the narratives were not rich enough to determine exactly what happened. But the vast majority of child deaths appeared to occur when a child or children found a gun in the home that was improperly stored. The children then play with the gun. Often it is explicitly reported that the shooter believes the gun is unloaded.

Reading the narratives allowed us to determine the number of cases in which hunting was mentioned in the text and the number of cases in which children were shot while visiting the homes of their friends. It might be useful if NVDRS abstractors added these as formal categories. Reading the cases was also important because we discovered that half of the original 16 cases of infants aged 0–1 had actually been sudden infant deaths (no firearms involved) and for some reason had been misclassified as unintentional firearm deaths.

This study has limitations. First, it provides data only about 16 states (for eight years). However, while the states were not randomly selected, they appear to be fairly representative of the United States, including states that are urban and rural, east and west coast, northern and southern, high-gun and low-gun. These states contain 26 % of the US child population, and had 26 % of the child gun deaths from all causes. We hope the NVDRS will continue to expand to cover all 50 states.

Second, while most cases were accompanied by a narrative which provided useful qualitative data, many narratives did not provide information on how the gun was accessed or exactly what happened. We felt that there were too many cases with missing data about the exact circumstances to create meaningful estimates of the prevalence of many behaviors (e.g., playing quick draw; thought the gun was unloaded). Third, there were fewer than 230 cases, so any confidence intervals for many estimates would be wide (e.g., the percentage of unintentional firearm deaths to children that are hunting-related; the relative number of child shooters aged 11–12 compared to shooters aged 13–14).

## Conclusions

 We estimate that over 100 children (aged 0–14) are accidentally shot and killed annually in the United States. The victims and shooters are overwhelmingly male. In the large majority of cases the victim has either shot himself or been shot by another child. In other-inflicted shootings, the shooter is usually a family member (most commonly a brother) or, particularly for children aged 13–14, a good friend.

The main problem is children shooting children, not adults shooting children. Nor is there a large problem of children shooting adults. Such findings indicate that the principal danger to children comes from the availability of firearms to children, their siblings, and their child friends.

## References

[CR1] Barber C, Hemenway D (2011). Too many or too few unintentional firearm deaths in official U.S. mortality data?. Accid Anal Prev.

[CR2] Barber C, Ozonoff V, Schuster M (1998). The Massachusetts weapon-related injury surveillance system. Am J Prev Med.

[CR3] Barber C, Azrael D, Hemenway D (2013). A truly national national violent death reporting system. Inj Prev.

[CR4] Cherry D, Runyan C, Butts J. A population-based study of unintentional firearm fatalities. Inj Prev 2001; 7:62–65 10.1136/ip.7.1.62 PMC1730683 11289538

[CR5] Connorton E, Miller M, Perry MJ, Hemenway D (2011). Mental health and unintentional injurers: results from the national co-morbidity survey replication. Inj Prev.

[CR6] Grossman DC, Reay DT, Baker SA (1999). Self-inflicted and unintentional firearm injuries among children and adolescents: the source of the firearm. Archives of Pediatric and Adolescent Medicine.

[CR7] Heins M, Kahn R, Bjordnal J (1974). Gunshot wound in children. Am J Public Health.

[CR8] Hemenway D, Barber CW, Gallagher SS, Azrael DR (2009). Creating a national violent death reporting system: a successful beginning. Am J Prev Med.

[CR9] Hemenway D, Barber C, Miller M. Unintentional firearm deaths: a comparison of other-inflicted and self-inflictedshootings. Acc Anal Prev. 2010; 42:1184–88. 10.1016/j.aap.2010.01.008 20441829

[CR10] Johnson RM, Lintz J, Gross D, Miller M, Hemenway D. Evaluation of the ASK campaign in two Midwestern cities. ISRN Public Health. 2012. Article ID 408124, 6 pages. doi:10.5402/2012/408124.

[CR11] Kalesan B, Villarreal MD, Keyes KM, Falea S. Gun ownership and gun culture. Injury Prevention*.* 2015. 041586. doi: 10.1136/injuryprev-2015-041586. [Epub ahead of print]. 10.1136/injuryprev-2015-041586 PMC4809774 26124073

[CR12] Lott J. Children and guns: the fear and the reality. National Review. 2013.

[CR13] Luo M, McIntire M. Children and guns: the hidden toll. New York Times. 2013.

[CR14] Moms Demand Action. Innocents Lost: A Year of Unintended Child Gun Deaths. 2014. http://everytownresearch.org/reports/innocents_lost/

[CR15] Ordog GJ, Wassweberger J, Schatz I, Owens-Collins D, Englsh K, Balasubramanium S (1988). Gunshot wounds in children under ten years of age. Am J Diseases in Children.

[CR16] Paulozzi LJ, Mercy J, Frazier L, Annest JL (2004). CDC’s national violent death reporting system: background and methodology. Inj Prev.

[CR17] Richardson EG, Hemenway D (2011). Homicide, suicide and unintentional firearm fatality: comparing the United States with other high-income countries, 2003. J Trauma.

[CR18] Schaechter J, Duran I, DeMarchena J, Lemard G, Villar ME (2003). Are ‘accidental’ gun deaths as rare as they seem? A comparison of medical examiner manner of death coding with an intent-based classification approach. Pediatrics.

[CR19] Vyrostek SB, Annest JL, Ryan GW (2004). Surveillance for fatal and non-fatal injuries—United States 2001. MMWR Surveillance Summary.

[CR20] Wintemute GJ, Teret SP, Kraus JF (1987). When children shoot children: eight-eight unintended deaths in California. JAMA.

